# New Analysis and Design of a RF Rectifier for RFID and Implantable Devices

**DOI:** 10.3390/s110706494

**Published:** 2011-06-24

**Authors:** Dong-Sheng Liu, Feng-Bo Li, Xue-Cheng Zou, Yao Liu, Xue-Mei Hui, Xiong-Fei Tao

**Affiliations:** Department of Electronic Science & Technology, Huazhong University of Science & Technology, Wuhan, 430074, China; E-Mails: dsliu@mail.hust.edu.cn (D.-S.L.); fbli1987@gmail.com (F.-B.L.); estxczou@hust.edu.cn (X.-C.Z.); yaoliuhust@gmail.com (Y.L.); huixuemei@gmail.com (X.-M.H.)

**Keywords:** radio frequency identification, passive transponders, diode-connected MOS transistor, rectifier, power conversion efficiency

## Abstract

New design and optimization of charge pump rectifiers using diode-connected MOS transistors is presented in this paper. An analysis of the output voltage and Power Conversion Efficiency (PCE) is given to guide and evaluate the new design. A novel diode-connected MOS transistor for UHF rectifiers is presented and optimized, and a high efficiency N-stage charge pump rectifier based on this new diode-connected MOS transistor is designed and fabricated in a SMIC 0.18-μm 2P3M CMOS embedded EEPROM process. The new diode achieves 315 mV turn-on voltage and 415 nA reverse saturation leakage current. Compared with the traditional rectifier, the one based on the proposed diode-connected MOS has higher PCE, higher output voltage and smaller ripple coefficient. When the RF input is a 900-MHz sinusoid signal with the power ranging from −15 dBm to −4 dBm, PCEs of the charge pump rectifier with only 3-stage are more than 30%, and the maximum output voltage is 5.5 V, and its ripple coefficients are less than 1%. Therefore, the rectifier is especially suitableto passive UHF RFID tag IC and implantable devices.

## 1. Introduction

The rapidly increasing range of applications of radio frequency identification (RFID) technology includes supply chain management, access control to buildings, public transportation, airport baggage handling, and express parcel logistics [1–3]. Use of a RFID system is a good approach for automated identification of products. The need for lower cost, higher data rates, and longer communication distances is increasing, while severe regulation of transmission power and bandwidth have to be met. RFID tags (or transponders) are often classified as passive or active. Passive tags are powered by an electromagnetic wave transmitted by the reader, while the active tag is powered by a battery. Passive tags have the advantages of low cost and long life. As the passive tag is remotely powered by a reader’s RF signal, it must be able to operate at very low power levels (∼μW) [1,3].

In 1999 the FCC allocated the Medical Implant Communication Service (MICS) band to the 402–405 MHz range. However, due to the low transmitted power of the MICS band (EIRP = 25 μW), this band cannot be used to power implanted system. This has motivated research on implantable transceiver architectures operating in other ISM bands, such as in the 902 to 928 MHz and 2.4 GHz ISM bands [4–6]. For example, [6] presented a wireless neural interface which harvests RF power from a standard commercial ultra-high frequency (UHF, 902 MHz–928 MHz) RFID reader.

The ultra-high-frequency (UHF) passive RFID tag or implantable device has to work at a considerably long distance from the transmitter (or reader). As the RF energy received by the tag (or implantable device) decreases rapidly with distance, the induced voltage across the tag antenna is often very small [6–9]. In order to obtain a high output voltage, an N-stage charge pump rectifier (also called a charge pump multiplier) is typically used [6,10,11]. Schottky diodes with low potential barrier are widely used for achieving a high output voltage and a high Power Conversion Efficiency (PCE), but Schottky diodes are not compatible with the standard CMOS process. Instead of Schottky diodes, diode-connected MOS transistors with very low threshold voltages are used in the N-stage voltage multiplier [7,8]. The weakness of using MOS transistors is that the threshold voltage is increased by the body effect. There were many publications regarding the design of RF rectifiers using diode-connected MOS transistors [7,8,12–17], however, there have been almost no technical papers regarding the design issue of providing a new diode-connected CMOS for substituting the Schottky diode. For example, the design strategy and efficiency optimization of UHF micro-power rectifiers using diode-connected MOS transistors with very low threshold voltage is presented in [7], but it didn’t solve the problems generated by the substrate bias effect of diode-connected MOS transistors.

In this paper, a novel diode-connected MOS transistor for replacing the Schottky diode was presented, and a high efficiency N-stage charge pump voltage rectifier circuit based on this new diode-connected MOS transistor was designed and implemented. Section 2 starts with the analysis of the N-stage rectifier using Schottky diodes. Section 3 describes the design and optimization of a new diode-connected MOS transistor. Section 4 gives simulation results for the N-stage rectifier using the novel diode-connected MOS transistor, and compares our new design with the traditional rectifier through these simulation results. Section 5 compares the simulation results with the theoretical analysis. Section 6 concludes our research efforts.

## 2. Analysis of an N-Stage Rectifier

**Figure 1.**
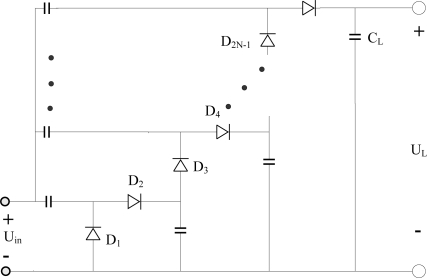
N-stage charge pump voltage rectifier.

Figure 1 shows an N-stage charge pump voltage rectifier consisting of a 2N peak value detector [1]. The input is assumed to be sinusoidal, with *U_in_* = *V*_0_ cos(*ω*_0_ *t*) [10]. To reduce the ripple voltage, the load capacitance C_L_ is assumed to be large enough, so as to make the time constant much bigger than the input signal cycle:

(1) $$\frac{I_{\text{L}}}{{CU}_{\text{L}}}\ll \omega_{0}$$

where U_L_ is output voltage, I_L_ is load current.

The equivalent circuit of the diode working at high frequency consists of two parts, which are an ideal diode and a parasitic capacitance C_D_, connected in parallel [11]. According to the V-I characteristics of the PN junction index model, the current of each diode is given by:

(2) $$\begin{array}{ll} i_{D} & =I_{s}(e^{v_{D}/V_{T}}-1)+C_{D}\frac{dV_{d}}{dt}\\ & =I_{s}\left[\text{exp}\left(\pm \frac{V_{0}}{V_{T}}\text{cos}(\omega_{0}t)-\frac{{\text{U}}_{\text{L}}}{2NV_{T}}\right)-1\right]\\ & +C_{D}\frac{dV_{d}}{dt} \end{array}$$

where, V_T_ is the thermal voltage, I_S_ is the reverse saturation current.

Because the capacitance doesn’t dissipate power, we ignore the parasitic capacitance when we study the large-signal characteristics. For the 2Nnd diode, we have:

(3) $$\begin{array}{ll} I_{d} & =I_{s}\left[\text{exp}\left(\frac{V_{0}\text{cos}(\omega_{0}t)-\frac{U_{L}}{2N}}{V_{T}}\right)-1\right]\\ & =I_{s}\left[\text{exp}\left(\frac{V_{0}\text{cos}(\omega_{0}t)}{V_{T}}\right)\text{exp}\left(\frac{-\frac{U_{L}}{2N}}{V_{T}}\right)-1\right]\\ & =I_{s}\left[{\left(\text{exp}(\text{cos}(\omega_{0}t))\right)}^{\frac{V_{0}}{V_{T}}}\text{exp}\left[\frac{-\frac{U_{L}}{2N}}{V_{T}}\right]-1\right] \end{array}$$

**Figure 2.**
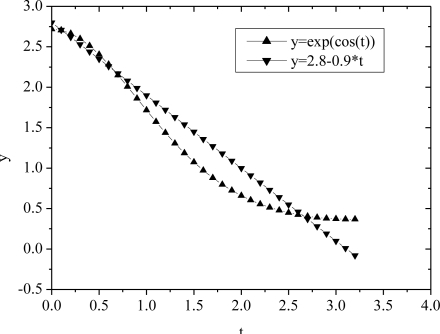
Linear approximation of exponential trigonometric function.

As the approximation shown in Figure 2, we assume *e*^cos(*ω*_0_*t*)^ ≈ 2.8 − 0.9*ω*_0_*t*, where $t\in [0,\frac{T}{2}]$, Equation (3) can be rewritten as:

(4) $$I_{d}=I_{s}[{\left(2.8-0.9\omega_{0}t\right)}^{\frac{V_{0}}{V_{T}}}\text{exp}\left(\frac{-\frac{U_{L}}{2N}}{V_{T}}\right)-1]$$

Integrating Equation (4) in one cycle gives:

(5) $$\begin{array}{l} I_{L}\times T=\int_{0}^{T}I_{d}dt\\ \;\;\approx 2\times I_{s}\int_{0}^{T/2}\left[{\left(2.8-0.9\omega_{0}t\right)}^{\frac{V_{0}}{V_{T}}}\text{exp}\left(\frac{-\frac{U_{L}}{2N}}{V_{T}}\right)-1\right]dt\\ \;\;=2\times I_{s}\text{exp}\left(\frac{-\frac{U_{L}}{2N}}{V_{T}}\right)\frac{{\left(2.8\right)}^{\frac{V_{0}}{V_{T}}+1}}{\left(\frac{V_{0}}{V_{T}}+1\right)0.9\omega_{0}}-I_{s}\times T \end{array}$$

So, the output voltage is:

(6) $$U_{L}=-2NV_{T}\text{ln}\left[\frac{5.652\times (I_{L}+I_{s})\left(\frac{V_{0}}{V_{T}}+1\right)}{2I_{s}\times {\left(2.8\right)}^{\frac{V_{0}}{V_{T}}+1}}\right]$$

**Figure 3.**
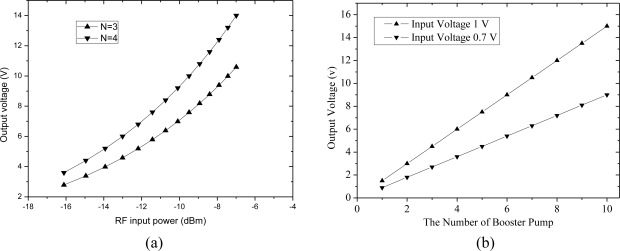
Output voltage curves of the charge pump rectifier **(a)** Output voltage with different input power **(b)** Output voltage with different number of charge pump stage.

Assuming that the load resistor R_L_ and I_S_ of Equation (6) are 10 KΩ and 1a A (1a = 10^−18^) respectively, the input impedance of the rectifier is 10 KΩ. The characteristic output voltage response is shown in Figure 3. Figure 3(a) shows that the output voltage increases approximately linearly with the RF input power. Figure 3(b) shows that the output voltage increases with rectifier stages N.

**Figure 4.**
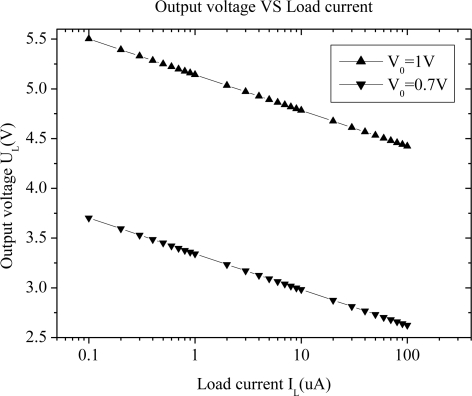
Output voltage of charge pump rectifier with different load currents.

Because the charge pump multiplier is usually used under light load conditions, the primary emphasis is placed on the law of output voltage with different output load current. As shown in Figure 4, output voltage decreases logarithmically with the load current increase.

Taking the strategy of linearly approximation as shown in Figure 2, to an N-stage rectifier, the power dissipated on all diodes in a cycle is:

(7) $$\begin{array}{l} P_{diode}=2\times N\times 2\int_{0}^{T/2}I_{s}\left[{\left(2.8-0.9\omega_{0}t\right)}^{\frac{V_{0}}{V_{T}}}\text{exp}\left(\frac{-\frac{U_{L}}{2N}}{V_{T}}\right)-1\right]\\ \;\;\;\;\;\;\;\;\;\;\;\;\;\;\;\;\;\;\;\;\;\;\;\;\;\;\;\;\times \left(V_{0}-0.64V_{0}\omega_{0}t-\frac{U_{L}}{2N}\right)dt\\ \;\;\;\;\;\;\;\;\;\;\;=4NI_{s}[\frac{0.791V_{0}}{\left(\frac{V_{0}}{V_{T}}+1\right)\left(\frac{V_{0}}{V_{T}}+2\right)\omega_{0}}\times \text{exp}\left(-\frac{U_{L}}{2NV_{T}}\right)\\ \;\;\;\;\;\;\;\;\;\;\;\;\;\;\;\;\;\;\;\;\;\;\;\;\;\times {\left(2.8\right)}^{\frac{V_{0}}{V_{T}}+2}+\frac{\left(V_{0}-\frac{U_{L}}{2N}\right)}{0.9\omega_{0}\left(\frac{V_{0}}{V_{T}}+1\right)}\times \text{exp}\left(-\frac{U_{L}}{2NV_{T}}\right)\\ \;\;\;\;\;\;\;\;\;\;\;\;\;\;\;\;\;\;\;\;\;\;\;\;\;\times {\left(2.8\right)}^{\frac{V_{0}}{V_{T}}+1}+\frac{3.15V_{0}}{\omega_{0}}-\left(V_{0}-\frac{U_{L}}{2N}\right)\frac{3.14}{\omega_{0}} \end{array}$$

The power dissipated on the load in one cycle is:

(8) $$P_{L}=\int_{0}^{T}I_{L}U_{L}dt=\frac{I_{L}U_{L}\times 2\pi }{\omega_{0}}$$

The power conversion efficiency is given by:

(9) $$PCE=\frac{P_{L}}{P_{L}+P_{diode}}$$

**Figure 5.**
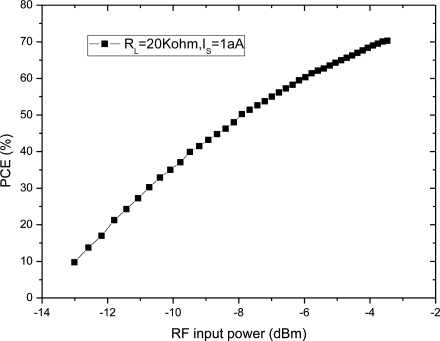
Power conversion efficiency with different input power.

Figure 5 shows the power conversion efficiency with different RF input power. PCE increases with the input power, but PCE tends to saturation as the rate of the increase gradually decreases.

## 3. Design and Analysis of a New Diode-Connected MOS Transistor

### 3.1. Design of Diode-Connected CMOS with Low Turn-On Voltage

When replacing a diode with a diode-connected MOS transistor, we have to assure that the new structure will turn on when it is forward-biased and will cut off when it is reverse-biased, so not only should the NMOS substrate connect to the lowest voltage, but also the PMOS substrate should connect to the highest voltage.

**Figure 6.**
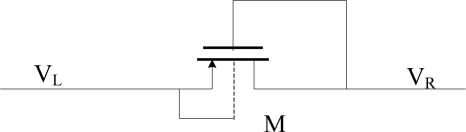
Diode-connected PMOS.

As shown in Figure 6, the substrate of a diode-connected PMOS is connected to its source. Then, when V_L_ is higher than V_R_, there will be a current from V_L_ to V_R_. It means that the diode turns on when it is forward-biased. On the other hand, when V_R_ is higher than V_L_, the voltage of drain is higher than that of the substrate, so the drain-body junction starts to conduct. It means that the diode doesn’t cut off when it is reverse-biased.

This can be solved by connecting the substrate of PMOS to the highest potential. But this will cause two problems in practice:

1. MOS transistor works in dynamic status, so it is difficult to decide the highest voltage.
2. Substrate bias effect will result in an increase of the threshold voltage, and make the turn-on voltage increase ultimately.

**Figure 7.**
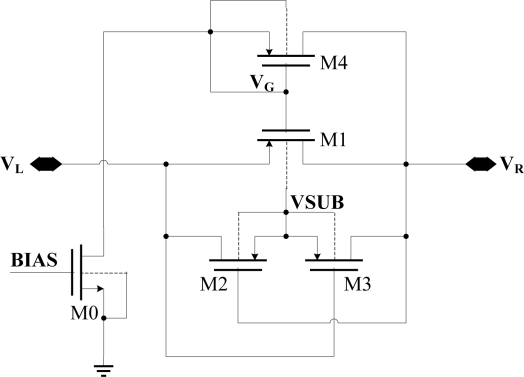
The schematic diagram of improved diode-connected CMOS.

The structure of improved diode-connected CMOS for replacing the Schottky diode is shown in Figure 7. The improved diode-connected CMOS could decrease the turn-on voltage and ensure that the substrate is connected to the highest voltage.

In Figure 7, M0 supplies an exiguity bias current via bias voltage BIAS. So VG will bias at a level of V_R_-V_TH_, where V_TH_ is the drain-body junction turn-on voltage. PMOS M2 and M3 are used to assure that the substrate of M1 is connected to the highest voltage. If V_L_ is higher than V_R_, M2 will turn on while M3 will cut off, and the potential of M1, M2 and M3’s substrate VSUB will rises up to V_L_. Otherwise, if V_R_ is higher than V_L_, M3 will turn on while M2 will cut off, and the potential of M1, M2 and M3’s substrate VSUB will rise up to V_R_. All these ensure that the substrate of M1, M2 and M3 is always connected to the highest potential, so that the drain-body junction is reverse-biased all the time.

When V_L_ is high, M1 works in the linear region and the turn-on resistance is small, so V_R_ can be as high as V_L_. On the other hand, when V_L_ is low, V_G_ is about V_R_-Vin. The absolute value of V_GD_ of M1 will be smaller than its threshold voltage, and M1 will cut off. In this way, we realize a diode which will turns on when it’s forward-biased with small voltage. In this diode, M0 is high voltage zero-threshold NMOS, whose threshold voltage is 0.31 V. M1, M2 and M3 are all high-voltage PMOS, whose threshold voltage is −0.92 V. The drain-body junction turn-on voltage of all these transistors is 0.7 V. It is notable that the absolute value of the threshold voltage V_TH_ of the PMOS is higher than the drain-body junction turn-on voltage V_th_. This new diode-connected CMOS has some advantages:

1. The problem of body-potential connecting is solved by the body-switching technique.
2. The control of M1’s gate voltage is realized by a simple bias circuit, which makes M1 be in the linear region rather than the saturation region when it is turned on. Therefore, this structure has a small forward voltage, which is applicable to a UHF RFID tag. When the voltage amplitude of the antenna in UHF RFID tag is small, high conversion efficiency and high output voltage are important to the rectifier of a UHF RFID tag.

### 3.2. Analysis and Optimization of Parasitic Effect for the New Diode-Connected CMOS

**Figure 8.**
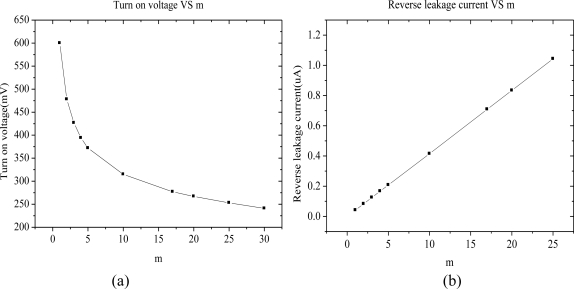
Characteristic curve of diode-connected CMOS **(a)** Turn-on voltage with different *m* **(b)** Reverse leakage current with different *m*.

M2 and M3 always work in the linear region, in order to connect the substrate voltage. So they don’t need big (W/L), actually, it’s 2/1 in this design. For M1, if the number of the multiple transistors is bigger, *i.e*., the W/L will be bigger, the turn-on resistance will be smaller, and then the turn-on voltage will be lower. However, bigger (W/L) brings a greater reverse leakage current, so we need a tradeoff between turn-on voltage, reverse leakage current and area. Figure 8 shows the different turn-on voltage (when conduction current is 100 μA) and different reverse leakage current (when reverse voltage is 1 V) with different number of parallel connection transistors. From Figure 8, we can see that the reverse leakage current gets greater with the increase of the number m of parallel connection transistors, and the reverse current is directly proportional to the m value. From the analysis above, 10 could be the probable number of multiple transistors. When the number is 10, the turn-on voltage is only 315 mV, and the reverse leakage current is 415 nA.

As shown in Figure 7, C_P_ = C_GS1_ is a relatively large parasitic capacitance, and is about 0.1 pf. When V_L_ changes, because of the coupling of CP, the gate voltage of M4 V_G_ will also change. Two situations are analyzed here under the condition that the RF input was a 900-MHz sinusoid.

1. If V_L_ decreases suddenly, the gate voltage of M4 V_G_ will decrease too. Then drain-body junction of M4 turns on, and C_P_ is charged to V_R_-V_TH_ (where V_TH_ is the turn-on voltage of M4’s drain-body junction) by the current of drain-body junction of M4. If V_G_ rises rapidly, the duration of low potential of V_G_ will be short, and then the conduction time of M1will be short too. It means that the reverse leakage current will be small. Besides, the charging rate depends on the current through the drain-body junction of M4. If the drain—body junction area is large, the turn-on current will be big, thus the charging rate will be high. Because the area of M4’s drain-body junction is directly related to the width of the transistor, the width of M4 should be as big as possible, so as to decrease the reverse leakage current. Assuming that the falling amplitude of V_L_ is V, the charging current to C_P_ through the drain-body junction I is constant, charging time is t, and the capacitance seen from V_G_ is C_X_, we have:
  (10) $$\frac{C_{P}C_{\text{x}}}{C_{P}+C_{\text{x}}}V=It$$
  If C_X_ is comparable with C_P_, V = 1, t is ten percent of the RF signal cycle, I will be 450 μA, which is easy to gain for a turn-on diode.
2. If V_L_ rises suddenly, the gate voltage of M4 V_G_ will rise too. The capacitance C_P_ discharges through M0 and M4. The shorter the discharging time is, the lower the turn-on voltage of the diode-connected MOS will be. The discharging time is determined by the current through M0 and M4. However, as long as V_G_ rises to no more than V_TH_ + V_th_, (where V_TH_ is the absolute value of PMOS threshold voltage, V_th_ is the turn-on voltage of drain-body junction,) M4 will cut off, the current will be close to 0, so that the discharging time depends mainly on the current I0 through M0. As the parasitic capacitance of MOS transistors, V_R_ changes little when it tends to stability. Therefore, V_R_ can be regarded as ground in the AC analysis. The parasitic capacitances of M1 are consisted the gate-drain parasitic capacitance Cgd1 and gate-source parasitic capacitance Cgs1. The effect of V_L_ on V_G_ is Cgs1 × V_L_/(Cgs1+Cgd1). Cgs1 and Cgd1 is nearly equal. So if the rise amplitude is V, V_G_ rise up about 1/2 V:
  (11) $$\frac{1}{2}V=I_{0}t$$

**Figure 9.**
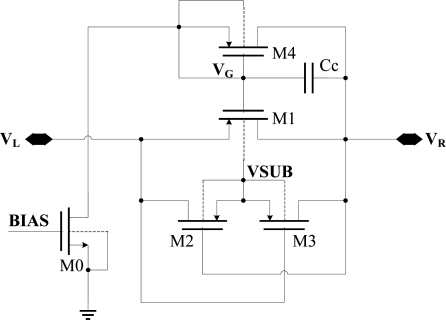
Schematic diagram of optimized diode-connected CMOS.

When V is 1, t is ten percent of the RF signal cycle, I0 is 450 μA. For bias transistor M0, I0 is huge and will make the power consumption much bigger, so it’s not advisable to increase I0. Fortunately, we could solve this problem by a compensatory capacitance Cc. With the Cc, the effect of V_L_ on V_G_ is Cgs1 × V_L_/(Cgs1+Cgd1+Cc), reducing the power consumption caused by coupling effect of parasitic capacitances. Actually we add a 10 pf capacitance between V_R_ and V_G_ in this design. The final schematic diagram of the optimized diode-connected MOS is shown in Figure 9.

## 4. Simulation Results and Discussions for N-Stage Rectifier

**Figure 10.**
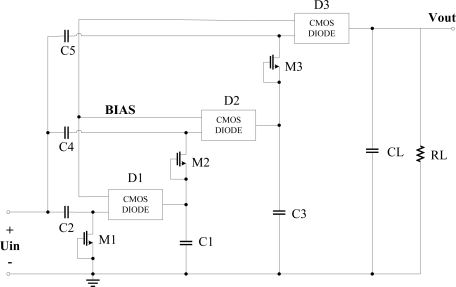
A improved 3-stage charge pump rectifier.

**Figure 11.**
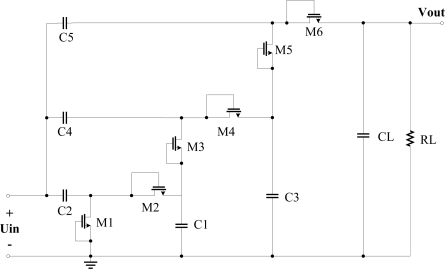
Traditional CMOS charge pump rectifier.

**Figure 12.**
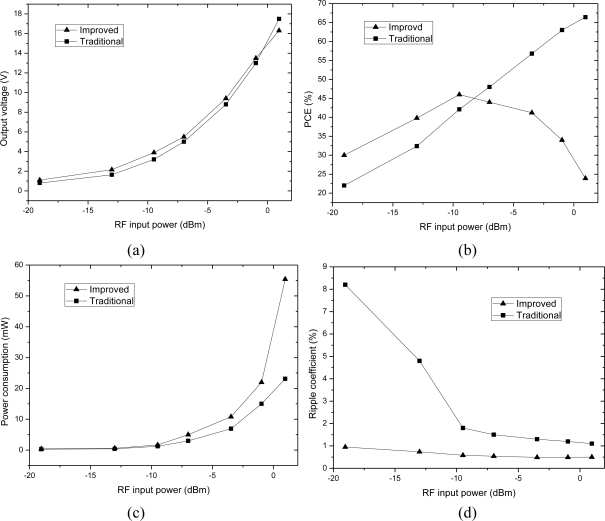
The curves of output voltage, PCE, power consumption and ripple coefficient with different the input power **(a)** Output voltages with different input power **(b)** PCEs with different input power **(c)** Power consumption with different input power **(d)** Ripple coefficients with different input power.

The N-stage rectifier using the novel diode-connected MOS transistor will be discussed in this Section. Figure 10 shows the structure of a 3-stage charge pump rectifier in which we use the optimized diode-connected MOS which is represented by a rectangle symbol. When the RFID tag enters an electromagnetic field, the input voltage is in negative half-cycle, current charges C2, C4 and C5 through NMOS M1, M2 and drain-body junction diode of M3. In positive half-cycle, current charges C1, C3 and CL through diode-connected MOS D1, D2 and D3. Compared with the traditional CMOS charge pump rectifier (shown in Figure 11), the proposed structure has the advantages of high PCE, low power consumption, high output voltage, and low ripple coefficient. When the RF input is a 900-MHz sinusoid, the two rectifiers are all designed and implemented in SMIC 0.18-μm 2P3M CMOS embedded EEPROM process. The simulation results of the key performance parameters is given in the same process, with the same load resistance 20 kΩ and the same input powers, compared with the traditional CMOS 3-stage charge pump multiplier. According to the key simulation data, we get the curves of output voltage, PCE and ripple coefficient with different the input powers as shown in Figure 12.

Figure 12 shows that the improved charge pump multiplier has a very small ripple coefficient, always below 1%. When the voltage amplitude is small, this novel charge pump multiplier has a distinct high output voltage and high PCE, compared with traditional rectifier. When the RF input is a 900-MHz sinusoid and the input power is −13 dBm (the input impedance of the rectifier is 10 KΩ, peak-to-zero amplitude is 1 V), conversion efficiency is 39.8%, and output voltage is 2.16 V, the ripple coefficient is below 1%. When the RF input is a 900-MHz sinusoid and the input power is −7 dBm, conversion efficiency is 46.9%, and output voltage is 5.76 V, the ripple coefficient is also below 1%. When the induced voltage across the antenna gets bigger, the improved rectifier’s power consumption increases markedly, and at the same time, the conversion efficiency decreases. The main reason can be explained by Figure 9: when the output voltage is very high, the reverse voltage on the drain-body junction diode of the bias transistor M0 is close to its reverse breakdown voltage, so the current of M0 can no longer be calculated in accordance with the mirror current. However, in practice, we can limit the output voltage smaller than 8 V through a regulator, so as to protect the chip.

## 5. Comparison between the Simulation Results and the Theoretical Analysis

**Figure 13.**
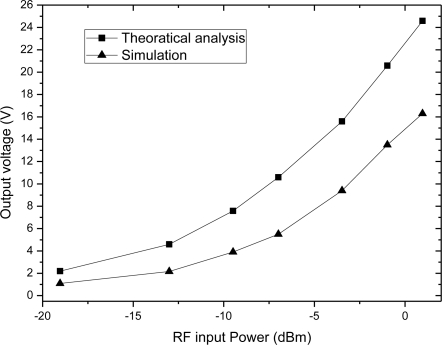
Comparison of the output voltage of the theoretical analysis and simulation results.

The comparison between Figure 12(a) and Figure 3(a) is plotted in the same figure, as shown in the Figure 13, which indicates their trends are identical, meaning the theoretical analysis in Figure 3(a) is correct.

**Figure 14.**
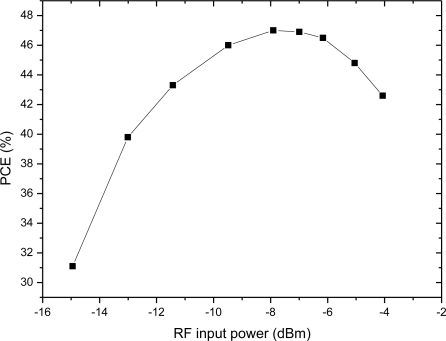
PCE of the 3-stage rectifier with different input power.

**Figure 15.**
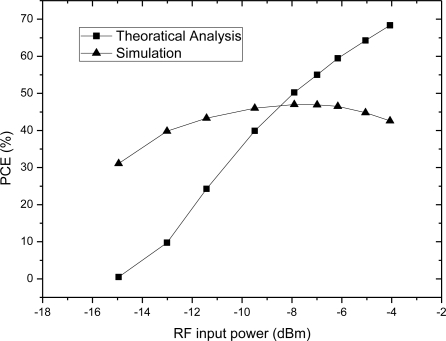
Comparison of the PCE of the theoretical analysis and simulation results.

The relationship between PCE and input voltage is simulated, when the RF input is a 900-MHz sinusoid and output load is 20 kΩ. According to the simulation results, we get the PCE of the charge pump multiplier curve with different input voltages as shown in Figure 14. The comparison between Figures 5 and 14 is plotted in the same figure, as shown in Figure 15, which indicates some differences. The simulation results show that PCE increases at the beginning of the input power and then decreases, whereas, the theoretical analysis in Figure 5 shows that PCE increases all the time and tends to saturation. The explanation is as follows: in this paper, we use a diode-connected NMOS and mirror NMOS. Their reverse voltage on drain-body junction rises with input voltage, their leakage current increases when the reverse voltage is close to its breakdown voltage, this bring superfluity power consumption. While in the theoretical model, we don’t take into account of breakdown model, which is the origin of difference between PCE curves.

## 6. Conclusions

The output voltage and PCE of the N-stage rectifier based on the equivalent model of diode are discussed. To optimize the output voltage and PCE, we analyze the disadvantages of the traditional diode-connected MOS rectifier, and propose a novel diode-connected MOS transistor for UHF micro-power rectifiers. The turn-on voltage of the novel structure is only 315 mV, and its reverse saturation leakage current is 415 nA. The proposed diode-connected MOS transistor has been successfully applied in passive RFID tags for PMOS bridge rectifiers [12] and N-stage charge pump rectifiers.

After that, a charge pump multiplier using the presented diode-connected MOS is designed and fabricated in the SMIC 0.18-μm three-metal two-poly mixed signal CMOS technology with embedded EEPROM process. Compared with a traditional rectifier, this circuit has higher PCE, higher output voltage and smaller ripple coefficient. When the RF input is a 900-MHz sinusoid signal with the power ranging from −15 dBm to −4 dBm, PCEs of the charge pump rectifier with only 3-stagesare stable between 30% and 47%, achieving much higher efficiency than charge pump rectifier designs reported in journals or conferences. For example, [16] also employed the SMIC 0.18-μm process, but the PCEs are stable between 26% and 36%. Such performances might open promising perspectives for the deployment of passive RFID tag IC and implantable device in standard CMOS process without Schottky diodes.
